# Estimating BDS-3 Satellite Differential Code Biases with the Single-Frequency Uncombined PPP Model

**DOI:** 10.3390/s23187900

**Published:** 2023-09-15

**Authors:** Jizhong Wu, Shan Gao, Dongchen Li

**Affiliations:** School of Geomatics Science and Technology, Nanjing Tech University, Nanjing 211800, China

**Keywords:** differential code bias, BeiDou-3 global navigation satellite system, single-frequency, precise point positioning, zero-mean condition

## Abstract

Differential Code Bias (DCB) is a crucially systematic error in satellite positioning and ionospheric modeling. This study aims to estimate the BeiDou-3 global navigation satellite system (BDS-3) satellite DCBs by using the single-frequency (SF) uncombined Precise Point Positioning (PPP) model. The experiment utilized BDS-3 B1 observations collected from 25 International GNSS Service (IGS) stations located at various latitudes during March 2023. The results reveal that the accuracy of estimating B1I-B3I DCBs derived from single receiver exhibits latitude dependence. Stations in low-latitude regions show considerable variability in the root mean square (RMS) of absolute offsets for satellite DCBs estimation, covering a wide range of values. In contrast, mid- to high-latitude stations demonstrate a more consistent pattern with relatively stable RMS values. Moreover, it has been observed that the stations situated in the Northern Hemisphere display a higher level of consistency in the RMS values when compared to those in the Southern Hemisphere. When incorporating estimates from all 25 stations, the RMS of the absolute offsets in satellite DCBs estimation consistently remained below 0.8 ns. Notably, after excluding 8 low-latitude stations and utilizing data from the remaining 17 stations, the RMS of absolute offsets in satellite DCBs estimation decreased to below 0.63 ns. These enhancements underscore the importance of incorporating a sufficient number of mid- and high-latitude stations to mitigate the effects of ionospheric variability when utilizing SF observations for satellite DCBs estimation.

## 1. Introduction

The Chinese BDS-3 stands as one of the four existing Global Navigation Satellite Systems (GNSSs). BDS-3 was initiated with the launch of its first satellite in 2017 and was subsequently declared operational in July 2020. BDS-3 represents the worldwide expansion of the regional BDS-2 system, which was established and operationalized between 2009 and 2012, ultimately commencing its operational service in December 2012 [1,2]. BDS-2 transmits B1I, B2I, and B3I signals, while BDS-3 extends its broadcasting capabilities to encompass B1I, B3I, B1C, B2a and B2b signals [3]. The augmentation in the quantity of BDS satellites, coupled with the diverse array of signals, presents a more formidable challenge when it comes to data processing.

Differential code biases (DCBs) in GNSS pertains to the discrepancies in time delays observed in code measurements within the satellite and receiver hardware channels, involving either the same or different frequencies. DCBs typically span from a few nanoseconds to several tens of nanoseconds, rendering a significant systematic error that necessitates attention in high-precision GNSS applications, such as SF PPP and ionospheric inversion [4,5]. Indeed, the signal propagation error induced by the ionosphere can lead to substantial discrepancies in satellite navigation and positioning, spanning from several meters to hundreds of meters. As part of GNSS data processing, the DCBs and ionospheric model parameters are closely lumped [6,7], and their estimation is often performed simultaneously [8,9,10]. Hence, any inaccuracies in the ionospheric modeling may propagate into errors in the estimation of satellite DCBs using this approach. The second approach involves utilizing external high-precision ionospheric products, such as the Global Ionospheric Map (GIM), to constrain the Vertical Total Electron Content (VTEC) parameters [11,12]. The satellite DCBs are then estimated based on the fixed VTEC values. However, the accuracy of this method heavily relies on the quality of the external ionospheric products [13,14]. Typically, GIM products offer an overall accuracy of only 2–8 TECU [8]. Therefore, this study will focus on estimating both the satellite DCBs and the ionospheric model parameters.

Securing highly precise ionospheric observables from BDS observation data is of utmost importance, serving as a crucial prerequisite to achieve accurate and reliable estimations of satellite DCB parameters [15]. The substantial increase in the number of BDS-3 satellites compared to BDS-2 provides a greater abundance of observations for conducting ionospheric inversion. This enhanced satellite constellation facilitates the attainment of high-precision estimates of VTEC and satellite DCBs through the utilization of BDS-3 signals. The increased spatial and temporal coverage afforded by BDS-3 enables more accurate and reliable ionospheric modeling, leading to improved VTEC and satellite DCBs estimation capabilities [16]. However, this advanced level of ionospheric modeling is heavily dependent on the utilization of datasets collected from a dense network of ground-based receivers, which provides a large volume of data for precise analysis and reconstruction of the ionospheric electron density distribution. The globally distributed IGS reference stations equipped with dual-frequency (DF) receivers were primarily established for geodetic applications. The current number of GNSS reference stations is insufficient to adequately support space–atmosphere studies, particularly for tropospheric and ionospheric sensing applications [17,18]. To achieve high-precision ionospheric modeling, a denser network of reference stations is required, which can lead to increased hardware costs. To address this issue, replacing expensive high-precision multi-frequency and multi-GNSS receivers with more affordable low-cost SF receivers can be advantageous in future planning. Leveraging the capabilities of cost-effective SF receivers makes it an attractive option to strike a balance between the need for dense station networks and the associated hardware costs. Nevertheless, the present dearth of relevant algorithms poses a challenge, creating an urgent demand for a cost-effective solution that can fulfill accuracy requirements. The development of such a low-cost solution would significantly enhance the feasibility of high-precision ionospheric modeling without incurring prohibitive hardware expenditures.

Several publications have demonstrated the potential of SF receivers for retrieving VTEC and satellite DCBs. The code-minus-carrier (CMC) combination method, utilizing SF pseudorange and carrier phase observations, has been employed to model the VTEC for high- and mid-latitude stations. However, it is noteworthy that the precision of VTEC estimation is significantly impacted by multipath and pseudorange noises [19]. Alternatively, the SF uncombined PPP model demonstrates its efficacy in simultaneously estimating satellite DCBs and VTEC parameters using SF receivers. Remarkably, when applied to GPS L1 data collected by a single receiver, this model achieves daily estimates of satellite DCBs with absolute deviations of less than 1 ns from their ground truth values [8]. In addition, VTEC parameters retrieved from the SF uncombined PPP model exhibit comparable accuracy to those achieved through the commonly used DF method [18] and carrier-to-code leveling (CCL) method [20].

Previous studies on the SF uncombined PPP model have primarily focused on its application for VTEC estimation, while research on satellite DCBs has been limited to the GPS system. The primary contribution of this study is the estimation of BDS-3 satellite DCBs using SF receivers, taking advantage of the sufficient number of observable BDS-3 satellites. Throughout the process, special attention is paid to address the conversion of zero-mean conditions, particularly when the availability of observable satellites for receivers exhibits non-stationary behavior. Furthermore, this study thoroughly investigates the influence of station latitude on the accuracy and stability of the estimated BDS-3 satellite DCBs. Additionally, it examines the significant benefits and advantages of utilizing multiple SF receivers for the precise estimation of BDS-3 satellite DCBs.

The paper is structured as follows: Section 1 is the Introduction, followed by Section 2 which introduces the methods and relevant theories for satellite DCBs estimation using the SF uncombined PPP model. Section 3 provides details about the experimental data used and the processing strategies employed. In Section 4, experimental evaluations are conducted using BDS-3 SF data from 25 IGS stations in March 2023. The BDS-3 satellite DCBs are estimated using both single receiver and multiple receivers based on the SF uncombined PPP. The accuracy of the estimated results is assessed by comparing them with the satellite DCB products provided by the Chinese Academy of Sciences (CAS). Section 5 summarizes the conclusions drawn from the experimental analysis.

## 2. Methods

### 2.1. Extraction of Ionospheric Observables Using the SF Uncombined PPP Model

The equations representing the raw code and carrier phase observations for the first frequency BDS measurements are as follows [21,22]:

(1) $$\left\{\begin{array}{l} P_{r,1}^{s}(i)=\rho_{r}^{s}(i)+m_{r}^{s}(i)Z_{r}(i)+dt_{r}(i)-dt^{s}(i)+ION_{r,1}^{s}(i)+b_{r,1}-b_{,1}^{s}+\varepsilon_{P}(i)\\ \Phi_{r,1}^{s}(i)=\rho_{r}^{s}(i)+m_{r}^{s}(i)Z_{r}(i)+dt_{r}(i)-dt^{s}(i)-ION_{r,1}^{s}(i)+\lambda_{1}N_{r,1}^{s}+\delta_{r,1}-\delta_{,1}^{s}+\varepsilon_{\Phi }(i) \end{array}\right.$$

In Equation (1), $P_{r,1}^{s}(i)$ and $\Phi_{r,1}^{s}(i)$ respectively denote the code and carrier phase observations from BDS satellite s to receiver r on the first frequency at epoch i; $\rho_{r}^{s}$ is the geometric distance of the transmitted signal from the satellite to the receiver; $m_{r}^{s}$ is the corresponding troposphere mapping function; $Z_{r}$ is the zenith tropospheric delay at receiver r; $dt_{r}$ and $dt^{s}$ represent the receiver clock offsets and the satellite clock offsets, respectively; $ION_{r,1}^{s}$ represents the slant ionospheric delay from the receiver to the satellite at the first frequency. $b_{r,1}$ and $b_{,1}^{s}$ respectively represent the code hardware delays of the receiver and satellite at the first frequency. $\lambda_{1}^{}$ denotes the carrier phase wavelength of the first frequency. $N_{r,1}^{s}$ is the carrier phase integer ambiguity at the first frequency. $\delta_{r,1}$ and $\delta_{,1}^{s}$ denote the carrier phase hardware delays for the receiver and the satellite. $\varepsilon_{P}^{}$ and $\varepsilon_{\Phi }^{}$ respectively represent the combination of observational noise and errors such as multipath effects in the code and carrier phase observations. Given the availability of high-precision priori known station coordinates and precise ephemeris data released from IGS analysis centers, it becomes feasible to compute the geometric distance at each epoch.

The precise satellite clock products $\hat{d}t^{s}$ provided by IGS analysis centers are derived through the ionosphere-free (IF) combination of code and carrier-phase observations. This means that the precise satellite clock products incorporate the IF combination of the satellite code hardware delays. It can be expressed as:

(2) $$\hat{d}t^{s}(i)=dt^{s}(i)+\frac{f_{1}^{2}}{f_{1}^{2}-f_{2}^{2}}b_{,1}^{s}-\frac{f_{2}^{2}}{f_{1}^{2}-f_{2}^{2}}b_{,2}^{s}$$

With the priori known precise clock products, Equation (1) can be expressed as:

(3) $$\left\{\begin{array}{l} P_{r,1}^{s}(i)-\rho_{r}^{s}(i)+\hat{d}t^{s}(i)=m_{r}^{s}(i)Z_{r}(i)+dt_{r}(i)+b_{r,1}+ION_{r,1}^{s}(i)+\frac{f_{2}^{2}}{f_{1}^{2}-f_{2}^{2}}\left(b_{,1}^{s}-b_{,2}^{s}\right)+\varepsilon_{p}^{}(i)\\ \Phi_{r,1}^{s}(i)-\rho_{r}^{s}(i)+\hat{d}t^{s}(i)=m_{r}^{s}(i)Z_{r}(i)+dt_{r}(i)-ION_{r,1}^{s}(i)+\lambda_{1}N_{r,1}^{s}+\delta_{r,1}-\delta_{,1}^{s}\\ \;\;\;\;\;\;\;\;\;\;\;\;\;+\frac{f_{1}^{2}}{f_{1}^{2}-f_{2}^{2}}b_{,1}^{s}-\frac{f_{2}^{2}}{f_{1}^{2}-f_{2}^{2}}b_{,2}^{s}+\varepsilon_{\Phi }^{}(i) \end{array}\right.$$

Equation (3) exhibits a rank deficiency function among the ionosphere, receiver clock offset, phase ambiguity parameters, and code hardware delay. This rank deficiency leads to non-unique solutions of Equation (3) [23,24]. To simplify the expression, let us assume that $\Delta P_{r,1}^{s}=P_{r,1}^{s}-\rho_{r}^{s}+\hat{d}t^{s}$ and $\Delta \Phi_{r,1}^{s}=\Phi_{r,1}^{s}-\rho_{r}^{s}+\hat{d}t^{s}$. After reparameterization and recombination, the resulting equation can be derived as follows:

(4) $$\left\{\begin{array}{l} \Delta P_{r,1}^{s}(i)=m_{r}^{s}(i)Z_{r}(i)+{\bar{dt}}_{r}(i)+{\bar{ION}}_{r,1}^{s}(i)+\varepsilon_{p}^{}(i)\\ \Delta \Phi_{r,1}^{s}(i)=m_{r}^{s}(i)Z_{r}(i)+{\bar{dt}}_{r}(i)-{\bar{ION}}_{r,1}^{s}(i)+{\bar{N}}_{r,1}^{s}+\varepsilon_{\Phi }^{}(i) \end{array}\right.$$

with

(5) $$\left\{\begin{array}{l} {\bar{dt}}_{r}(i)=dt_{r}(i)+b_{r,1}\\ {\bar{ION}}_{r,1}^{s}(i)=ION_{r,1}^{s}(i)+\frac{f_{2}^{2}}{f_{1}^{2}-f_{2}^{2}}\left(b_{,1}^{s}-b_{,2}^{s}\right)\\ {\bar{N}}_{r,1}^{s}=\lambda_{1}N_{r,1}^{s}+\delta_{r,1}-\delta_{,1}^{s}-b_{r,1}+\frac{f_{1}^{2}+f_{2}^{2}}{f_{1}^{2}-f_{2}^{2}}b_{,1}^{s}-\frac{2f_{2}^{2}}{f_{1}^{2}-f_{2}^{2}}b_{,2}^{s} \end{array}\right.$$

In Equation (4), there is still a rank deficiency of size one between the re-parametrized ${\bar{dt}}_{r}$, ${\bar{ION}}_{r,1}^{s}$ and ${\bar{N}}_{r,1}^{s}$, which means that they cannot be estimated independently. Through recombining the biased receiver clock offset parameters into the variation amount of the receiver clock offset of subsequent epochs relative to the first epoch receiver clock offset, and recombining the parameters in Equation (4), the resulting equation is a full-rank SF uncombined PPP model equation:

(6) $$\left\{\begin{array}{l} \Delta P_{r,1}^{s}(1)=m_{r}^{s}(1)Z_{r}(1)+{\bar{\bar{ION}}}_{r,1}^{s}(1)+\varepsilon_{P}(1)\\ \Delta \Phi_{r,1}^{s}(1)=m_{r}^{s}(1)Z_{r}(1)-{\bar{\bar{ION}}}_{r,1}^{s}(1)+{\bar{\bar{N}}}_{r,1}^{s}+\varepsilon_{\Phi }(1)\\ \Delta P_{r,1}^{s}(i)=m_{r}^{s}(i)Z_{r}(i)+{\bar{\bar{dt}}}_{r}(i)+{\bar{\bar{ION}}}_{r,1}^{s}(i)+\varepsilon_{P}(i)\;(i\geq 2)\\ \Delta \Phi_{r,1}^{s}(i)=m_{r}^{s}(i)Z_{r}(i)+{\bar{\bar{dt}}}_{r}(i)-{\bar{\bar{ION}}}_{r,1}^{s}(i)+{\bar{\bar{N}}}_{r,1}^{s}+\varepsilon_{\Phi }(i)\;(i\geq 2) \end{array}\right.$$

with

(7) $$\left\{\begin{array}{l} {\bar{\bar{dt}}}_{r}(i)={\bar{dt}}_{r}(i)-{\bar{dt}}_{r}(1)\\ {\bar{\bar{ION}}}_{r,1}^{s}(i)={\bar{ION}}_{r,1}^{s}(i)+{\bar{dt}}_{r}(1)=ION_{r,1}^{s}(i)-\frac{f_{2}^{2}}{f_{1}^{2}-f_{2}^{2}}(b_{,2}^{s}-b_{,1}^{s})+{\bar{dt}}_{r}(1)\\ {\bar{\bar{N}}}_{r,1}^{s}={\bar{N}}_{r,1}^{s}+2{\bar{dt}}_{r}(1) \end{array}\right.$$

In Equation (7), $DCB_{2,1}^{S}=b_{,2}^{s}-b_{,1}^{s}$, the estimable parameter ${\bar{\bar{ION}}}_{r,1}^{s}$ can be represented as:

(8) $${\bar{\bar{ION}}}_{r,1}^{s}(i)=ION_{r,1}^{s}(i)-\frac{f_{2}^{2}}{f_{1}^{2}-f_{2}^{2}}DCB_{2,1}^{S}+{\bar{dt}}_{r}(1)$$

The estimable ionospheric parameter ${\bar{\bar{ION}}}_{r,1}^{s}$, known as the ionospheric observables, encompasses the slant ionospheric delay, satellite DCB and ${\bar{dt}}_{r}(1)$. Note that ${\bar{dt}}_{r}(1)$ represents the combined value of the first epoch receiver clock offset and the receiver code hardware delay at the first frequency. Due to the expected stability of the receiver code hardware delay under normal environmental conditions [8,18], ${\bar{dt}}_{r}(1)$ is treated as a constant during the daily processing. However, it is essential to be aware that if the data collection is interrupted for any reason, a gap will appear between subsequent and preceding results. This gap occurs because a new receiver clock offset is introduced as the first epoch receiver clock offset in such cases.

### 2.2. Conversion of Zero-Mean Conditions

Currently, the majority of analysis centers utilize the zero-mean condition as the constraint for satellite DCBs [25,26]. This condition assumes that the sum of all satellite DCBs is equal to zero. However, over longer observation periods, satellites may experience failures or replacements, leading to non-stationary availability of observable satellites for users. Consequently, satellite DCBs computed under the zero-mean constraint are considered as relative values. In situations where comparisons of satellite DCBs are required or when assessing the stability of satellite DCBs over extended time periods, it becomes necessary to convert satellite DCBs computed under different constraint conditions to a common constraint condition. This conversion ensures consistency and facilitates accurate comparisons and analyses of satellite DCBs.

If a particular GNSS comprises a total of *n* satellites in normal operation, the zero-mean condition C1 for all satellites can be expressed as follows:

(9) $$\sum_{i=1}^{n}DCB_{C1}^{s}=0$$

where s represents the PRN of the satellite observed by the receiver.

Assuming the receiver observes or utilizes *m* satellites from the satellite navigation system in actual observation, the new zero-mean condition C2 for all the observed satellites can be expressed as:

(10) $$\sum_{i=1}^{m}DCB_{C2}^{s}=0$$

Considering the two aforementioned zero-mean conditions, there exist *m* common satellites. The sum of the satellite DCBs for these *m* satellites under condition C1 can be expressed as follows:

(11) $$\sum_{i=1}^{m}DCB_{C1}^{s}=d\;(d\neq 0)$$

In order to transform the satellite DCBs from condition C1 to condition C2, the process involves subtracting a constant bias d/m from all satellite DCBs under condition C1. This transformation yields the satellite DCBs under condition C2, which can be expressed as follows:

(12) $$\left\{\begin{array}{l} {\hat{DCB}}_{C1\to C2}^{s}=DCB_{C1}^{s}-\frac{d}{m}\\ \sum_{i=1}^{m}{\hat{DCB}}_{C1\to C2}^{s}=0 \end{array}\right.$$

### 2.3. Ionospheric Modeling

**Figure 1:**
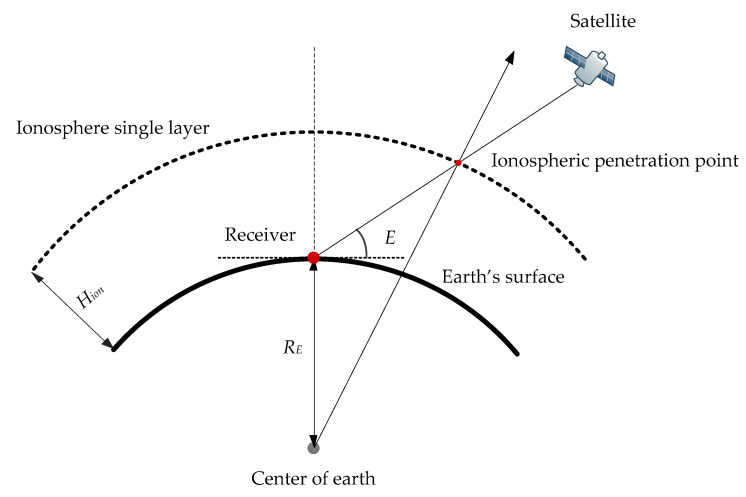
Schematic of thin-layer ionosphere model.

The distribution of electron density in the ionosphere is known to be non-uniform. To simplify the study of the ionosphere, a common simplification is made by assuming that all electrons in the ionosphere are concentrated on an infinitesimally thin layer at a specific height. This simplified representation, referred to as the thin-layer ionospheric model, is depicted in Figure 1.

The point at which the line-of-sight direction from the receiver intersects with the thin layer of the ionosphere is known as the ionospheric penetration point (IPP). The height of the thin layer ionosphere is typically set within the range of 350–450 km in ionospheric modeling. Under the assumption of the thin layer ionospheric model, the mapping function MF(z) facilitates the conversion of the ionospheric STEC along the line of sight between the satellite and the penetration point into the ionospheric VTEC at the penetration point. This conversion can be represented as follows:

(13) $$MF(z)=\frac{STEC}{VTEC}={\left[1-\frac{R_{e}^{2}\cos^{2}E}{{\left(R_{e}+H_{ion}\right)}^{2}}\right]}^{-\frac{1}{2}}$$

where $R_{e}^{}$ denotes the mean Earth radius, which is set to 6371 km; E is the elevation angle of the satellite with respect to the receiver; and $H_{ion}$ represents the ionospheric thin layer height which is taken as 450 km [19,20].

The utilization of the generalized triangular series function model enables simulation of the diurnal variation in local ionosphere total electron content (TEC) [12]. By employing this model to represent single-station ionosphere VTEC and applying a zero-mean condition, it becomes possible to separate the ionospheric VTEC and satellite DCBs from the ionospheric observations (Equation (8)). This separation can be resolved by solving the following equation:

(14) $$\left\{\begin{array}{l} VTEC(\varphi_{IPP},h_{IPP})=\sum_{a=0}^{2}\sum_{b=0}^{2}\left[E_{ab}{\left(\varphi_{IPP}-\varphi_{R}\right)}^{a}\cdot h_{IPP}{}^{b}\right]+\sum_{k=1}^{4}\left[C_{k}\cos(k\cdot h_{IPP})+S_{k}\sin(k\cdot h_{IPP})\right]\\ h_{IPP}=\frac{2\pi (t_{i}-14)}{24}\\ {\bar{\bar{ION}}}_{r,1}^{s}=\frac{A}{f_{1}^{2}}\times MF(z)\times VTEC(\varphi_{IPP},h_{IPP})-\frac{f_{2}^{2}}{f_{1}^{2}-f_{2}^{2}}DCB_{2,1}^{s}+\bar{d}t_{r}(1)\\ \sum_{i=1}^{N}DCB^{i}=0 \end{array}\right.$$

In the equation, $\varphi_{IPP}$ and $\varphi_{R}$ denote the geomagnetic latitude of the IPP and the receiver, respectively; $h_{IPP}$ is a function related to the local time $t_{i}$ of the IPP, $E_{ab}$, $C_{k}$ and $S_{k}$ are the coefficients of the generalized triangular series function model to be estimated, and N is the total number of satellites observed by the receiver.

**Figure 2:**
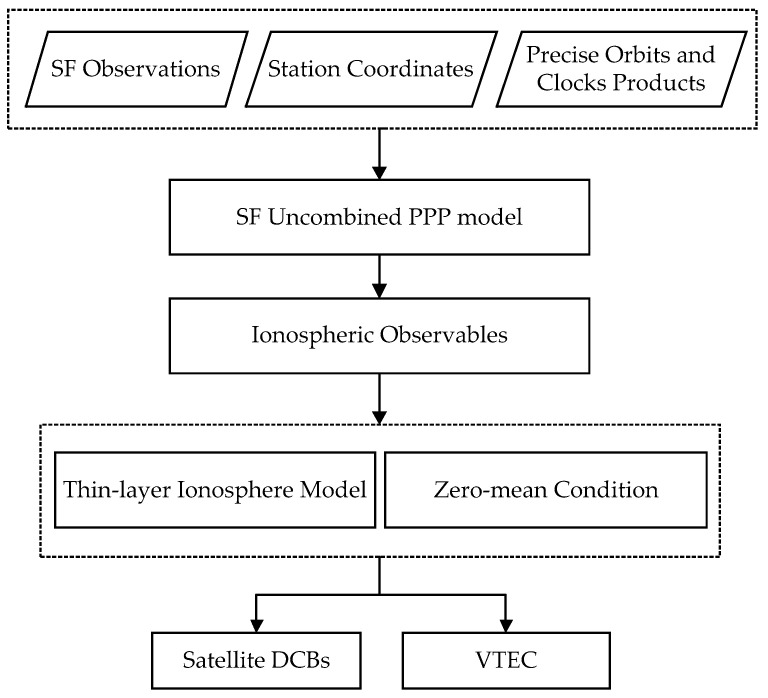
Schematic of satellite DCBs estimation using the SF uncombined PPP model.

The specific procedure for estimating satellite DCBs using the SF uncombined PPP model is depicted in Figure 2. The process begins with the estimation of ionospheric observables utilizing the SF uncombined PPP approach. This involves using the SF BDS B1 observations, along with external precise ephemeris and clock offset products, as well as the high-precision priori known station coordinates. Subsequently, ionospheric observables are utilized in conjunction with a thin-layer ionosphere model and the zero-mean condition to jointly estimate the satellite DCBs and VTEC parameters.

## 3. Experimental Data and Processing Strategy

In this study, the estimation of satellite DCBs incorporates the simultaneous modeling of the ionosphere using the generalized triangular series function model. However, it is important to note that the time-varying nature of the ionosphere exhibits unique characteristics at different latitudes [27,28]. Taking into consideration the potential influence of ionospheric on the accuracy of the estimated satellite DCBs, it becomes imperative to carry out comprehensive analyses across various time periods and latitude distributions. This approach will provide valuable insights into the temporal and spatial variations and aid in improving the overall precision of the DCB estimates.

**Figure 3:**
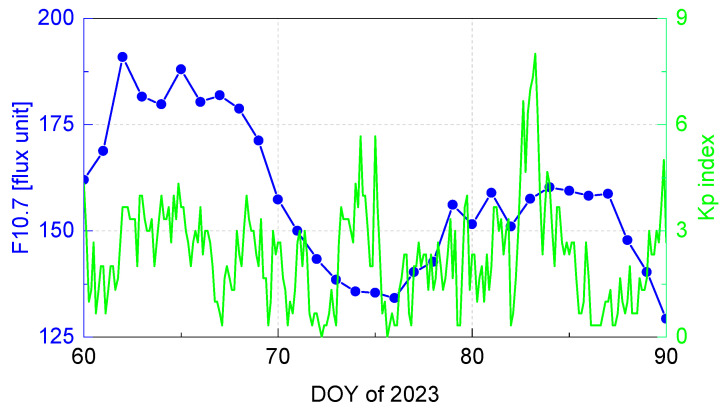
F10.7 and Kp indexes over the period of March 2023 (DOY 60~90).

Considering the experiment’s objectives and prerequisites, the dataset used in this study consists of observations gathered from 25 globally distributed IGS stations over the period of March 2023 (DOY 60~90). This temporal span possesses a significant representative nature due to its coverage of diverse geomagnetic and solar conditions, which are depicted in Figure 3. Within this temporal scope, the solar activity level proxy F10.7, displays a variation ranging from 129 to 190 solar flux units. Notably, the Kp index, which serves as an indicator of geomagnetic activity, surges to a value exceeding 6 on DOY 82 and 83, underscoring the occurrence of a severe geomagnetic storm during this period.

Notably, only the observations on the B1 frequency of each station are utilized in the analysis. Table 1 provides an overview of the data and processing strategy employed in this study for estimating satellite DCBs.

It is essential to emphasize that the BDS-3 satellite DCBs obtained using the SF uncombined PPP model are closely tied to the precise clock products employed in the analysis. The choice of dual-frequency IF combinations varies among different analysis centers. Most analysis centers typically utilize the B1/B3 IF combination to generate precise clock products, while some analysis centers may use the B1/B2 IF combination instead. This distinction in the selection of the IF combination can impact the specific BDS satellite DCB estimates. In this study, the precise clock products provided by Wuhan University (WUM) are employed, and the strategy for generating WUM products has shifted from B1I/B2I to B1I/B3I since DOY 1, 2019 [30]. As a result, when referring to satellite DCBs within the scope of this experiment, it specifically pertains to the C2I-C6I satellite DCBs.

The estimated DCBs were subsequently compared to the DCB products provided by CAS. Notably, the DCB products from CAS are computed using the pseudorange-leveled carrier-phase approach. This approach incorporates a generalized triangular series function to model the ionosphere for each individual station while simultaneously estimating the DCBs [31].

## 4. Experimental Results and Analysis

### 4.1. Satellite DCBs Estimated by SF Data from Single Station

During the period of March 2023 (DOY 60~90), satellite DCBs were estimated individually at each station using the SF uncombined PPP model, employing BDS-3 B1 observations gathered from 25 stations. The absolute offset of DCB for each satellite was calculated on a daily basis, serving as an assessment of the accuracy and performance of the model employed in this study. To evaluate the overall accuracy and reliability of the estimated DCBs, the daily RMS of the absolute offsets was computed at each station. The RMS value provides a quantitative measure of the accuracy and stability of the estimated DCBs on a daily basis.

**Figure 4:**
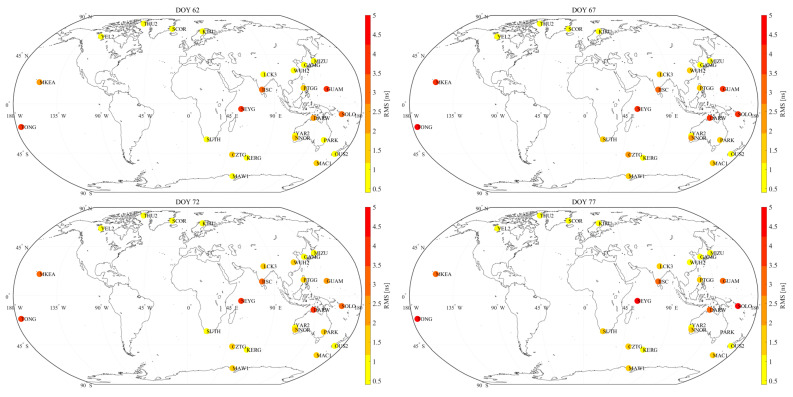

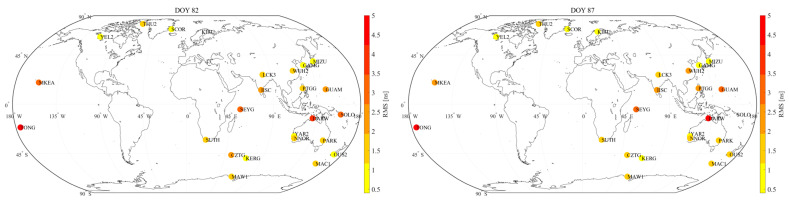
The RMS values of the satellite DCB estimates on six selected days.

Due to space limitations, we only present the results from six selected days in March 2023. The selected days, namely DOY 62, 67, 72, 77, 82, and 87, were chosen to represent different temporal points within the month. Figure 4 displays the station distribution and the corresponding RMS values of satellite DCB estimates for the selected days. The magnitude of the RMS values is portrayed through a color scheme, with intensifying shades of red indicating higher RMS values. This visualization provides an overview of the distribution and magnitude of the RMS values across different latitudes.

Notably, the results shown in Figure 4 exhibit minimal variation across different days. The visual depiction clearly demonstrates a noticeable pattern: the RMS values of the satellite DCB estimates are higher for low-latitude (30° N–30° S) stations, indicating lower precision in these estimates. Conversely, the RMS values of the satellite DCB estimates from mid- and high-latitude IGS stations are lower, indicating higher precision.

This discrepancy can be ascribed to the heightened activity and dynamic behavior of the ionosphere in low-latitude regions. When comparing with the mid- and high-latitude ionosphere, the low-latitude ionosphere presents greater spatial gradients and more intensive temporal variations, posing challenges in accurately characterizing the ionospheric variations within this region [28]. Consequently, such ionospheric complexities adversely affect the performance of ionosphere modeling, leading to a degradation in the accuracy of estimated satellite DCBs in this particular region. The variation in RMS values of the satellite DCB estimates across different latitudes underscores the impact of ionospheric variability on the precision and dependability of these estimations.

**Figure 5:**
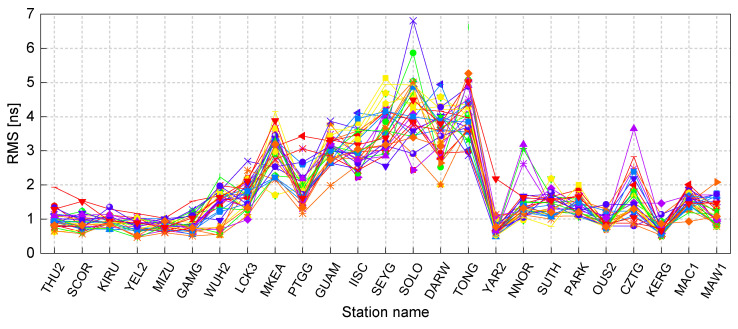
The RMS values of the satellite DCB estimates for all stations over 30 days.

To provide a more comprehensive assessment of the accuracy of the estimated satellite DCBs, the RMS values for all stations over 30 consecutive days are presented in the form of a line graph in Figure 5. This representation employs various line colors to differentiate between individual days throughout the duration of the experiment. The line graph highlights the variations in the RMS values of the satellite DCB estimates across different stations. It is important to note that the station names displayed on the horizontal axis of Figure 5 are arranged in descending order from north to south based on the latitude value of each station. To be precise, the first six stations, namely THU2 to GAMG, are situated at latitudes higher than 30° N, while the last nine stations, namely YAR2 to MAW1, are located at latitudes lower than 30° S. The remaining stations fall within the latitude range of 30° N to 30° S.

The observed discrepancies in the accuracy of estimated satellite DCBs across different stations, despite utilizing the same estimation method, are evident from the results. Notably, the estimated satellite DCBs display significantly more fluctuations in the low latitude regions. In addition, there are several stations that frequently demonstrate RMS values exceeding 3 ns, particularly those situated within the latitude range of 25° N to 25° S. These stations include MKEA, PTGG, GUAM, IISC, SEYG, SOLO, DARW, and TONG. This pattern of higher RMS values at these low-latitude stations indicates lower accuracy in the estimated satellite DCBs. The RMS values at low-latitude stations display a significant range of variation, spanning from 1.31 ns to 6.81 ns. This wide range emphasizes the relatively lower reliability of satellite DCB estimates at low-latitude stations. However, it is important to note that on specific days, such as DOY 88 for NNOR and CZTG, and DOY 61 for LCK3, the RMS values of the satellite DCB estimates exceeded 2 ns even at mid- and high-latitude stations. Overall, the RMS values at mid- and high-latitude stations remained below 2 ns, indicating a higher level of accuracy compared to the estimates at low-latitude stations.

Based on the repeatability of RMS values across different days, Figure 4 illustrates that, among the mid- and high-latitude stations, there is a higher level of consistency in RMS values for stations located in the Northern Hemisphere, as opposed to those in the Southern Hemisphere. Specifically, the RMS values of stations in the Northern Hemisphere remain around 1 ns, whereas the RMS values of stations in the Southern Hemisphere display a wider range. This feature suggests that the accuracy of the satellite DCB estimates is relatively more stable and consistent in the Northern Hemisphere. Conversely, in the Southern Hemisphere, there may be more variability in the accuracy of the estimates. This phenomenon indicates that ionospheric irregularities are more pronounced in the Southern Hemisphere compared to the Northern Hemisphere.

In general, the RMS value of satellite DCB estimates using SF data from a single station is expected to be better than 1 ns, particularly in the Northern Hemisphere. From this perspective, SF receivers are comparable to DF receivers for satellite DCBs estimation. However, ionospheric variability can introduce additional challenges and complexities in ionospheric modeling, potentially leading to increased variability in the accuracy of satellite DCB estimates in the Southern Hemisphere.

### 4.2. Satellite DCBs Estimated by SF Data from Multiple Stations

The preceding section has demonstrated the capability of a single SF receiver to estimate satellite DCBs. Nevertheless, it is crucial to acknowledge that the accuracy of these estimates may vary, primarily due to ionospheric variability. To address this challenge and achieve more robust outcomes, the incorporation of SF data from multiple receivers becomes imperative.

**Figure 6:**
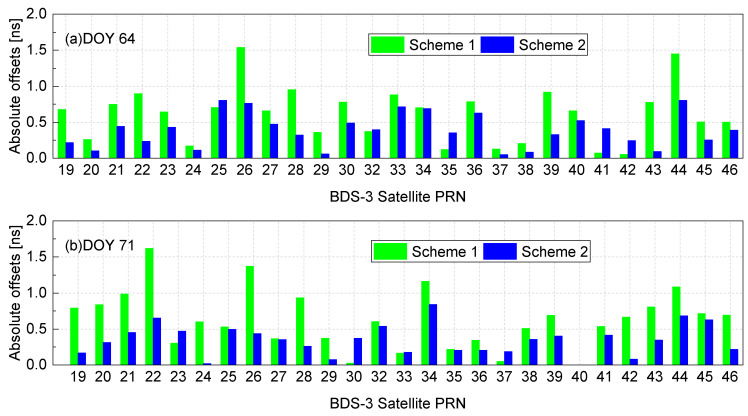
The absolute offsets for all BDS-3 satellites in two schemes on DOY 64 and 71, 2023.

In this section, two schemes, namely scheme 1 and scheme 2, are proposed for estimating satellite DCBs using data from multiple receivers. In scheme 1, the satellite DCBs are derived from a total of 25 stations, as depicted in Figure 5. This scheme utilizes observations from all 25 stations to estimate the satellite DCBs. In scheme 2, a subset of stations is selected by excluding eight low-latitude stations. The remaining 17 stations are used to estimate the satellite DCBs. In both scheme 1 and scheme 2, the satellite DCBs are estimated for each individual station. For each satellite, the DCBs obtained from the selected stations are averaged to derive the final value of satellite DCBs. This final satellite DCBs are compared to the CAS DCB products to calculate the absolute offset. As an illustrative example, the absolute offsets for all BDS-3 satellites on DOY 64 and 71, 2023, are provided in Figure 6a,b, respectively. Note that the BDS-3 PRN40 was not included in the WUM precision product on DOY 71, resulting in the absence of the corresponding result for this particular satellite in Figure 6b.

On DOY 64, scheme 2 resulted in a reduction in the absolute offsets for 22 out of the 27 BDS satellites compared to scheme 1. The RMS of the absolute offsets for scheme 1 was 0.72 ns, while for scheme 2 it was reduced to 0.45 ns. This reduction in RMS indicates an improvement in the accuracy of the satellite DCB estimates when using scheme 2. Similarly, on DOY 71, excluding the low-latitude stations in scheme 2 led to a reduction in the absolute offsets for 22 out of the 26 BDS-3 satellites. The RMS of the absolute offsets for scheme 1 was 0.76 ns, whereas for scheme 2 it was reduced to 0.41 ns. Once again, scheme 2 resulted in improved estimation accuracy for satellite DCBs. Consistently across both days, scheme 2 yielded absolute offset values for all BDS-3 satellites below 1 ns, indicating closer agreement with the CAS DCB product. This contrast supports the conclusion that excluding low-latitude data leads to improved accuracy in the estimation of satellite DCBs. These experimental results demonstrate that scheme 2 enhances the estimation accuracy of satellite DCBs, as evidenced by the reduced absolute offsets and lower RMS values.

**Figure 7:**
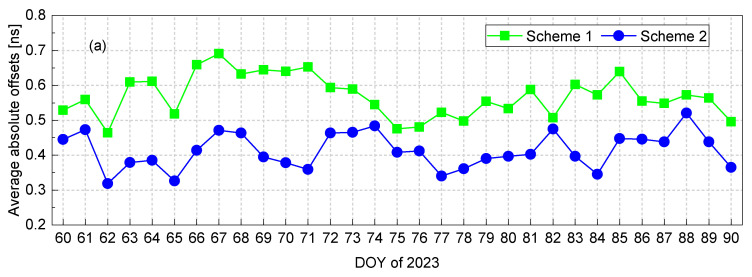

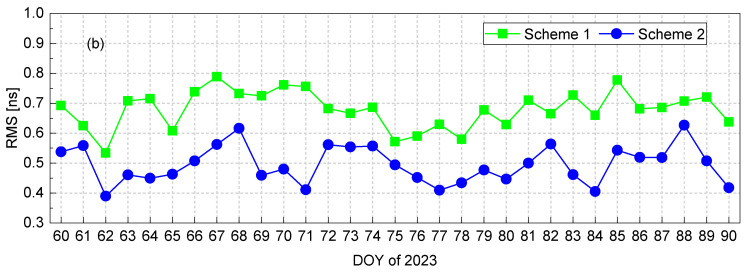
The daily average and RMS values of the absolute offsets in the estimated satellite DCBs throughout the experiment’s duration.

Figure 7 reveals insights into the performance of scheme 1 and scheme 2 for the estimation of BDS-3 satellite DCBs throughout the experiment’s duration.

According to the data presented in Figure 7a, when considering scheme 1, the average daily absolute offsets in the satellite DCB estimates remained below 0.7 ns, with a monthly average of 0.57 ns. Interestingly, scheme 2, which excluded data from low-latitude stations, consistently exhibited daily mean absolute offsets below 0.5 ns, resulting in a monthly average of 0.41 ns, except for the experimental result on DOY 88. Further analyzing Figure 7b, it can be observed that in scheme 1, the daily RMS of absolute offsets in March remained below 0.8 ns, with a monthly average of 0.68 ns. On the other hand, scheme 2 consistently displayed daily RMS values below 0.63 ns, leading to a notable monthly average of 0.49 ns. These findings substantiate a notable refinement in the accuracy of DCB estimates, especially when excluding data from low-latitude stations, thereby minimizing the influence of ionospheric variability prevalent in such regions on ionospheric modeling.

In summary, the implementation of scheme 2 has resulted in significant improvements in the accuracy of the estimated satellite DCBs compared to the performance in scheme 1. This multiple station approach has proven to be crucial in enhancing the precision and reliability of satellite DCBs estimation. By leveraging the cost-effectiveness of SF receivers, a denser ground receiver network is attainable, mitigating accuracy discrepancies in ionospheric modeling across various regions and, thereby, improving the comprehensive precision and reliability of satellite DCBs estimations. Embracing observations from a network of monitoring stations yields a more exhaustive and representative dataset, minimizing the impact of localized ionospheric effects and elevating the overall quality of the satellite DCB estimates.

## 5. Conclusions

This study endeavors to estimate BDS-3 satellite DCBs utilizing the SF uncombined PPP model. The experimental setup involved the utilization of BDS-3 B1 observations from a network of 25 globally distributed stations during the month of March 2023. Independently estimated satellite DCBs at each station unveiled latitude-dependent variations in accuracy. The RMS of the absolute offsets in satellite DCB estimation spanned from 1.31 ns to 6.81 ns at stations situated in low-latitude regions, suggesting relatively lower stability. In contrast, stations located in mid- and high-latitude regions predominantly exhibited RMS values below 2 ns, indicating improved stability and accuracy compared to their low-latitude counterparts. Moreover, the stations located in the Northern Hemisphere exhibit a relatively higher level of consistency in the RMS values compared to those in the Southern Hemisphere.

To mitigate the influence of regional characteristics on ionospheric modeling and address the disparities in modeling accuracy across different regions, a strategy is employed where ionospheric observables acquired from a number of selected stations are used to obtain a final satellite DCB value for each satellite. When utilizing estimates from all 25 stations, the RMS of the absolute offsets in satellite DCBs estimation remained consistently below 0.8 ns throughout March 2023, with a monthly average of 0.68 ns, exhibiting better accuracy compared to the satellite DCBs estimation by single receiver. By excluding 8 low-latitude stations and utilizing the data from the remaining 17 stations to estimate the final satellite DCBs, a notable improvement in accuracy was observed. The RMS of the absolute offsets in satellite DCBs estimation was further reduced to below 0.63 ns, with a monthly average of 0.49 ns. This process helps to minimize the biases and uncertainties introduced by localized ionospheric effects, ensuring a more balanced and reliable estimation of the satellite DCBs. This improvement in accuracy highlights the effectiveness and necessity of excluding low-latitude stations in enhancing the accuracy of satellite DCB estimates, especially when employing SF receivers for this purpose.

The SF receiver offers a cost-effective solution for estimating satellite DCBs; however, the SF uncombined observations is susceptible to the influence of ionospheric variability, which substantially impacts the accuracy of satellite DCB estimates. To address this issue, it is strongly advised to employ observations acquired from an extensive network of uniformly distributed monitoring stations situated in mid- and high-latitude regions, particularly when aiming to accurately estimate satellite DCBs. Given the cost-effectiveness advantage of SF receivers, it indeed becomes feasible to augment the density of the ground network by deploying affordable SF receivers. This strategic approach not only fulfills the demands of space–atmosphere studies but also serves as a substantial enhancement to observational capabilities for various other GNSS applications.

## Figures and Tables

**Table 1:** Data and Processing Strategies.

Items	Strategies
Filtering method	Least square filter
Processing mode	Day-by-day processing
Observations	BDS-3 B1 code and phase observations
Cut-off mask angle	15°
Precise products (orbits and clocks)	Wuhan University (WUM)
Tropospheric zenith wet delay	Random walk estimation
Hydrostatic delay	Empirical model [29]
Tropospheric projecting function	GMF/NMF
Slant ionospheric delay	Estimated as the white noise process
Priori station coordinates	IGS weekly station coordinates product
Antenna corrections	PCO/PCV parameters provided by igs14.atx
Earth rotation parameters	IGS weekly product
Tidal corrections	Solid Earth tide, ocean tide, and pole tide
Phase wind-up effect	Empirical model
C1 observations	IGS P1-C1 DCB corrections
Ambiguity parameter	Float ambiguity

## Data Availability

The IGS stations observation files, precise orbits and clocks products can be downloaded from the IGS Data Center of Wuhan University (ftp://igs.gnsswhu.cn/pub/ (accessed on 1 May 2023)). The CAS DCB products are found at CDDIS (https://cddis.nasa.gov/archive/gnss/products/bias/ (accessed on 1 May 2023)). The Kp index and F10.7 index data were obtained from the GFZ German Research Centre for Geosciences (https://kp.gfz-potsdam.de/en/data (accessed on 3 September 2023)).
